# Evaluating large language models as agents in the clinic

**DOI:** 10.1038/s41746-024-01083-y

**Published:** 2024-04-03

**Authors:** Nikita Mehandru, Brenda Y. Miao, Eduardo Rodriguez Almaraz, Madhumita Sushil, Atul J. Butte, Ahmed Alaa

**Affiliations:** 1 University of California, Berkeley, 2195 Hearst Ave, Warren Hall Suite, 120C, Berkeley, CA USA; 2 Bakar Computational Health Sciences Institute, University of California San Francisco, San Francisco, CA USA; 3 Neurosurgery Department Division of Neuro-Oncology, University of California San Francisco, 400 Parnassus Avenue, 8th floor, RM A808, San Francisco, CA USA; 4 Department of Epidemiology and Biostatistics, University of California San Francisco, 400 Parnassus Avenue, 8th floor, RM A808, San Francisco, CA USA; 5 Department of Pediatrics, University of California San Francisco, San Francisco, CA USA

**Keywords:** Health care, Medical research

## Abstract

Recent developments in large language models (LLMs) have unlocked opportunities for healthcare, from information synthesis to clinical decision support. These LLMs are not just capable of modeling language, but can also act as intelligent “agents” that interact with stakeholders in open-ended conversations and even influence clinical decision-making. Rather than relying on benchmarks that measure a model’s ability to process clinical data or answer standardized test questions, LLM agents can be modeled in high-fidelity simulations of clinical settings and should be assessed for their impact on clinical workflows. These evaluation frameworks, which we refer to as “Artificial Intelligence Structured Clinical Examinations” (“AI-SCE”), can draw from comparable technologies where machines operate with varying degrees of self-governance, such as self-driving cars, in dynamic environments with multiple stakeholders. Developing these robust, real-world clinical evaluations will be crucial towards deploying LLM agents in medical settings.

The release of ChatGPT, a chatbot powered by a large language model (LLM), has brought LLMs into the spotlight and unlocked opportunities for their use in healthcare settings. Med-PaLM 2, Google’s medical LLM, was found to consistently perform at a human expert level on medical examination questions scoring 85%^1^. While this model, part of Google’s family of foundation models known as MedLM, are fine-tuned for the healthcare industry, even large LLMs trained on openly available information from the Internet, not just biomedical information, have immense potential to improve and augment clinical workflows^2–4^. For instance, the Generative Pre-trained Transformer-4 (GPT-4) model can generate summaries of physician–patient encounters from transcripts of conversations^5^, achieve a score of 86% on the United States Medical Licensing Examination (USMLE)^6^, and create clinical question-answer pairs that are largely indistinguishable from human-generated USMLE questions^7^. These early demonstrations of GPT-4 and other LLMs on clinical tasks and benchmarks suggest that these models have the potential to improve and automate aspects of clinical tasks.

However, the emergent capabilities of LLMs have significantly expanded their potential beyond conventional, standardized clinical natural language processing (NLP) tasks that primarily revolve around text processing and question answering. Instead, there is a growing emphasis on utilizing LLMs for more complex physician- and patient-facing tasks that may involve multi-step information synthesis, use of external data sources, high-level reasoning, or even simulation of clinical text and conversations^8,9^.

In these scenarios, LLMs should not be viewed as models of language, but rather as intelligent “agents” that have internal planning capabilities that allow them to perform complex, multi-step reasoning or interact with tools, databases, other agents, or external users to better respond to user requests^9,10^. Here, we discuss how LLM agents can be used in clinical settings, and challenges to the development and evaluation of these approaches.

## Development of LLM agents for clinical use

LLM agents can be developed for a variety of clinical use cases by providing the LLM access to different sources of information and tools, including clinical guidelines, databases containing electronic health records, clinical calculators, or other curated clinical software tools^9,10^. These agents can respond to user requests by autonomously identifying and retrieving relevant information, or performing multi-step analyses to answer questions, model data, or produce visualizations. Different agents can also even interact and collaborate with each other in “multi-agent” settings to identify or check proposed solutions to difficult problems, or to model medical conversations and decision-making processes^11^.

Healthcare systems are already adopting LLMs capable of powering clinical agents; for instance, UC San Diego Health is working to integrate GPT-4 into MyChart, Epic’s online health portal, to streamline patient messaging^12^. Patients also leverage publicly available chatbots (such as ChatGPT) to better understand medical vocabulary from clinical notes, and some medical centers are exploring a “virtual-first” approach where LLMs assist in patient triaging^13,14^. When connected to additional sources of information and tools, the versatility and adaptability of clinical agents make them well-suited in supporting both routine administrative tasks as well as clinical decision support.

## Clinical simulations using agent-based modeling (ABM)

To evaluate the utility and safety of LLM-based chatbots as agents in these applications, we suggest the use of benchmarks that are not confined to traditional, narrowly-scoped assessments based on NLP benchmarks, which consist of predetermined inputs and ground-truths. Instead, approaches from agent-based modeling (ABM)^15^ can be used to create a simulated environment for effective evaluation of LLMs agents. ABM is a computational framework that simulates the actions and interactions of autonomous agents to provide insights into system-level behavior and outcomes. This approach has been used in health policy, biology, and the social sciences to conduct studies that simulate health behaviors and the spread of infectious diseases^16,17^.

ABM has also been used to evaluate autonomous agents in the domain of self-driving cars^18^. In this field, simulations of real-world environments containing road obstacles, traffic signals, other cars, and pedestrians can be used to evaluate and refine the behaviors of autonomous vehicle agents as they encounter these different elements^19^. Similarly, by simulating the clinical settings where LLM agents may be deployed, including patient-physician interactions and hospital processes, we can use an ABM approach to evaluate how an LLM agent may interact with users, which tools or data an LLM employs to carry out user requests, and points of failure that lead to erroneous outputs or downstream errors.

Interestingly, patients and physicians can also be simulated as LLM agents in ABM environments. Previous research has demonstrated the feasibility of employing LLMs to create “interactive simulacra” that replicate human behavior^9–11^. To develop these high-fidelity simulations, data on physician and patient behavior can be derived from real-world electronic health records or clinical trial data, ideally with validation from multiple hospital systems, and encompassing diverse patient populations. De-identified datasets (e.g., MIMIC-IV, UCSF Information Commons) or federated learning approaches can be used to help protect patient privacy^20,21^.

## Evaluating agent-based simulations using an AI-SCE framework

Similar to standards and regulations for the autonomous driving industry, identifying robust clinical guidelines and what constitutes a successful interaction for healthcare LLM agents will be crucial towards fulfilling the long-term goals of patients, providers, and other clinical stakeholders. In medical education, there has been a shift from assessing students using standardized testing which evaluates shallow clinical reasoning to modern curricula which increasingly use Objective Structured Clinical Examination (OSCE)^22^. These exams assess a student’s practical skills in the clinic, including the ability to examine patients, take clinical histories, communicate effectively, and handle unexpected situations. Google recently developed Articulate Medical Intelligence Explorer (AMIE), a research AI system for diagnostic medical reasoning and conversations, which was evaluated against the performance of primary care physicians (PCPs) in the style of an OSCE^23^.

Current benchmarks for clinical NLP, including MedQA (USMLE-style questions) and MedNLI, test if two clinical statements logically follow each other and are often also derived from standardized tests or curated clinical text. This information; however, is not a sufficient metric because it fails to capture the full range of capabilities demonstrated by clinical LLM agents^24,25^. As a result, we call for the development of Artificial Intelligence Structured Clinical Examinations (AI-SCEs) that can be used to assess the ability for LLMs to aid in real-world clinical workflows. These AI-SCE benchmarks, which may be derived from difficult clinical scenarios or from real-world clinical tasks, should be created with input from interdisciplinary teams of clinicians, computer scientists, and medical researchers. OSCEs typically consist of long lists of processes or diagnoses students are graded on. Similarly, AI-SCE benchmarks would extend beyond traditional computer science metrics, such as BLEU or ROUGE scores, that often do not account for semantic meaning, and would draw from preexisting multi-turn benchmarks^26^.

The AI-SCE format should be used to evaluate both the outputs of high-fidelity agent simulations, and intermediate steps that capture the agent’s reasoning process, tool usage, data curation, or interactions with other agents or external users. Thus, a valuable contribution of these agents is their ability to provide interpretability throughout the decision-making process, as opposed to at the final step^27^. These evaluations can also capture how systematic addition or removal of LLM agents affects overall outcomes. These evaluations should be used to inform guardrails for clinical LLMs, which have been developed for general-purpose models to constrain their behavior^28^.

One added complexity of assessing agents using an AI-SCE format is the complicated nature of many clinical tasks, where there may not be perfect concordance with individual human evaluators. We emphasize the continued need for a panel of human evaluators, and the importance of testing agent outcomes on external datasets. We also recognize the importance of post-deployment monitoring to ensure data distribution shifts do not occur over time, and to mitigate bias in model performance^25^. Furthermore, randomized control trials (RCTs) should be conducted to compare how well these simulation environments capture real-world settings, as well as the real-world impact of LLM agents in augmenting clinical workflows.

As LLMs evolve and demonstrate increasingly advanced capabilities, their involvement in clinical practice will extend beyond limited text processing tasks^29^. In the near future, it may become necessary to shift our benchmarks from static datasets to dynamic simulation environments and transition from language modeling to agent modeling. Drawing inspiration from fields such as biology and economics could be beneficial for future LLM research and development for clinical applications.
